# The interactions of *Candida albicans* with gut bacteria: a new strategy to prevent and treat invasive intestinal candidiasis

**DOI:** 10.1186/s13099-023-00559-8

**Published:** 2023-06-27

**Authors:** Fei Wang, Zetian Wang, Jianguo Tang

**Affiliations:** Department of Trauma-Emergency & Critical Care Medicine, Shanghai Fifth People’s Hospital, Fudan University, 128 Ruili Road, Shanghai, 200240 China

**Keywords:** *Candida albicans*, Fungal-bacterial interactions, Gut microbiota, Polymicrobial infection

## Abstract

**Background:**

The gut microbiota plays an important role in human health, as it can affect host immunity and susceptibility to infectious diseases. Invasive intestinal candidiasis is strongly associated with gut microbiota homeostasis. However, the nature of the interaction between *Candida albicans* and gut bacteria remains unclear.

**Objective:**

This review aimed to determine the nature of interaction and the effects of gut bacteria on *C. albicans* so as to comprehend an approach to reducing intestinal invasive infection by *C. albicans*.

**Methods:**

This review examined 11 common gut bacteria’s interactions with *C. albicans*, including *Escherichia coli*, *Pseudomonas aeruginosa*, *Acinetobacter baumannii*, *Enterococcus faecalis*, *Staphylococcus aureus*, *Salmonella spp.*, *Helicobacter pylori*, *Lactobacillus spp.*, *Bacteroides spp.*, *Clostridium difficile*, and *Streptococcus spp*.

**Results:**

Most of the studied bacteria demonstrated both synergistic and antagonistic effects with *C. albicans*, and just a few bacteria such as *P. aeruginosa*, *Salmonella spp.*, and *Lactobacillus spp.* demonstrated only antagonism against *C. albicans*.

**Conclusions:**

Based on the nature of interactions reported so far by the literature between gut bacteria and *C. albicans*, it is expected to provide new ideas for the prevention and treatment of invasive intestinal candidiasis.

## Background

Invasive candidiasis is a common fungal infection that majorly affects immunocompromised individuals and it is an important cause of death in severely ill patients [1]. It can be caused by several *Candida spp.*, which are common commensal organisms of the skin and gut microbiota, and disruptions in the cutaneous and gastrointestinal barriers; however, the prevalence of these organisms varies considerably depending on geographical location [2]. This disease presents as an entire spectrum of diseases, ranging from fungemia to deep-seated candidiasis and to septic shock with multiorgan failure, with an associated mortality rate of > 70% [2, 3]. Clinically, the treatment of systemic fungal infections often requires large doses and long courses of antifungal drug treatments; nonetheless, the mortality rate of severe patients remains as high as 40–50% [4], which makes the treatment of invasive fungal infections a major clinical challenge. *C. albicans*, which colonizes the gut, is the most dominant pathogen of invasive candidiasis. In a disordered system of the intestinal microenvironment and microecology, *C. albicans* takes the opportunity to multiply excessively, the transition from the yeast phase to the pathogenic mycelial phase, express adhesion molecules, release *C. albicans* toxin, destroy the gut mucosal barrier, and invade the blood, resulting in sepsis and multiple organ insufficiency, both of which are fatal [5]. Considering that the gut microbiota is the most important line of defense to maintain the intestinal epithelial barrier and block the invasion of intestinal *C. albicans* [6], understanding the multiple interactions occurring between the gut bacteria and *C. albicans* is a very promising research field. In recent years, an increasing number of studies on the human gut microbiota have presented several discoveries, which continue to constantly refresh our understanding of this field. Several past reviews have attempted to summarize the nature of interactions between *C. albicans* and gut bacteria as either antagonistic or synergistic; however, several of the reported interactions are not a single pattern, rather they are both synergistic and antagonistic. Therefore, we have provided a comprehensive summary of the interactions between *C. albicans* and some valuable gut bacteria in an attempt to achieve better insight into the current state of research in this field as well as to develop new ideas for designing strategies toward the prevention and treatment of invasive intestinal candidiasis.

## Main text

## The adaptations of *C. albicans* in the gut

The rapid adaptation of *C. albicans* to the gut microenvironment is closely associated with its colonization ability. The formation of hyphae is directly associated with the virulence of *C. albicans*, which represents a greater destructive power; hence, it is difficult for *C. albicans* in its hyphal form to establish a good intestinal symbiotic homeostasis [7–11]. Thus, the filamentation of *C. albicans* is inhibited to colonize the gut. In this process, Efg1 plays an important role, although it is affected by hypoxia and the host’s immune status [11, 12]. In addition, *C. albicans* can transform itself into an opaque (a/α), grey, and gastrointestinally induced transition (GUT) cell to adapt to the changing gastrointestinal environment [8, 13, 14].

In addition to changing their phenotypes for establishing intestinal symbiotic homeostasis, *C. albicans* has to adapt to other gut conditions, including the carbon source problem.

Glucose is the preferred carbon source for *C. albicans*, but the amount of glucose varies in different parts of the gut. For example, the glucose level in the large intestine, especially the colon, is low as most of the glucose is absorbed by the small intestine before it enters the large intestine. Thus, *C. albicans* is often forced to use alternative carbon sources that allow it to survive at the ecological sites with or without glucose [15–19]. Moreover, *C. albicans* can sense amino acids through the Ssy1p-Ptr3p-Ssy5p (SPS) sensor and hydrolyze activated transcription factors Stp1 and Stp2 to utilize amino acids as a carbon source [20–23]. Rtg3 and Sfp1 can help *C. albicans* to adjust the utilization of lactic acid and glucose according to environmental changes [24]. Fatty acids can be used as a substitute carbon source for *C. albicans* through β-oxidation [25]. Moreover, because gluconeogenic and glyoxylate cycle enzymes are unaffected by ubiquitin-mediated decomposition of metabolites, both the glycolysis and gluconeogenesis pathways remain active in *C. albicans*, such that *C. albicans* can utilize multiple carbon sources simultaneously [16, 26–30]. This flexible carbon-assimilation strategy of *C. albicans* enhances its ability to colonize and infect mammalian hosts [27]..

While living in the gut, *C. albicans* often face the issue of scarce resources or excessive ingestion. To better absorb iron from the environment, *C. albicans* has developed a safe mechanism for iron uptake and utilization whereby it absorbs iron using several different strategies, such as acquiring RBC-derived iron and inducing the expression of high-affinity iron permease gene *CaFTR1* and ferrichrome-type siderophores [31–34]. Meanwhile, to avoid toxicity caused by excessive iron, *C. albicans* possesses the transcription factor Sfu1 that inhibits iron uptake, various transcription factors that control iron permeability (such as plasma membrane-related Ftr1 and Ftr2 and vacuole-related Fth1 and Fth2) [34–36], and a variety of iron oxidases [36–39] .

Copper uptake is also a crucial factor. The key aspect in the copper uptake by *C. albicans* is ScCc2, a copper-transporting P-type ATPase that has an important role in iron transport [40]. It can express transcription factors Mac1 and Ctr1, which promote copper absorption, and can also avoid toxic effects caused by excessive copper accumulation through its unique plasma membrane structure and the activation of the copper resistance assay gene *CRD1* [41–45].

In addition to iron and copper, zinc is essential for the growth and biofilm formation of *C. albicans*. To obtain zinc from the host, *C. albicans* possess a complete set of regulatory modes. Past studies have demonstrated that under the mediation of Zrt1 and Zrt2, *C. albicans* can obtain zinc from the host environment with the help of pH-regulated antigen 1 (Pra1), a secreted zinc scavenger (“zincophore”), and a secreted aspartic protease Sap6. The ZnT-type transporter Zrc1 then stores zinc in the vacuole [46–49]. Zrt2 also ensures zinc uptake in an acidic environment, and Zap1 regulates the zinc homeostasis in *C. albicans.* [47, 49].

In addition to the problem of carbon sources and trace elements, *C. albicans* faces pressure from the guts’ physical and chemical environment, but *C. albicans* can resist them through specific signaling pathways [50, 51]. For example, resistance to the osmotic pressure and oxidative stress can be promoted through the Hog1-mediated MAP kinase pathway [52–54]; the gene *CAP1*, which codes for a bZip transcription factor of the AP-1 family, drives transcriptional responses to oxidative stress [55–57]; Mkc1- and Cek1-mediated MAP kinase pathways promote *C. albicans* resistance to cell wall stress [51, 58, 59]; transcription factors Cta4 and Hsf1 respond to the intestinal nitrosation stress and heat shock [60–62]. In addition, *C. albicans* can produce prostaglandin PGE2 from host-derived arachidonic acid to potentiate fungal fitness by acting on the fungi themselves and/or host tissue phagocytes to improve the ability of *C. albicans* to evade killing by phagocytes, thereby creating more favorable conditions for colonization [63]. These stress responses are essential for the survival of *C. albicans* in the gut, and, if the key stress responses get inactivated, colonization and virulence of *C. albicans* are significantly reduced.

While *C. albicans* struggles to adapt to the gut environment, these adaptability changes also inevitably reshape its original virulence, invasiveness, ability to defend itself against the host’s immune system, and susceptibility to antifungal drugs. The colonization ability of this fungal strain is driven by a complex regulatory network that connects metabolism, morphogenesis, stress adaptation, and cell wall remodeling, thereby affecting its symbiotic and infection-causing ability [64]..

## *C. albicans* and *gut bacteria*

### *C. albicans* and *Escherichia coli*

**Fig. 1:**
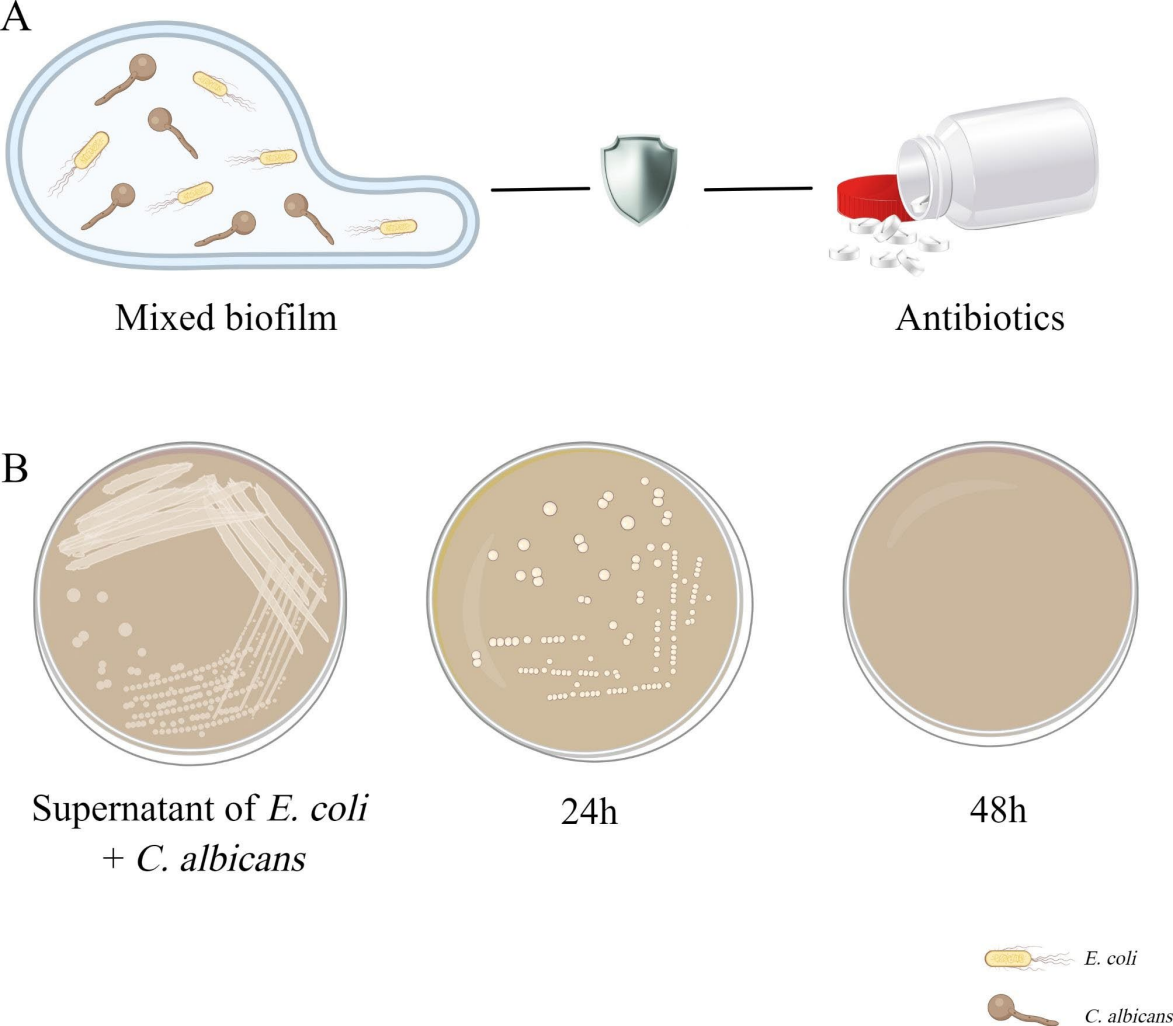
The interaction between *C. albicans* and *E. coli*. **(A)** Mixed biofilms formed by *C. albicans* and *E. coli* are more beneficial for resistance to antibiotics. **(B)** The supernatant of *E. coli* biofilm can restrain *C. albicans*

*E. coli* is a gram-negative bacterium and one of the major bacterial species found in the gastrointestinal tract of warm-blooded animals. This species consists of harmless, symbiotic bacterial and different pathogenic variants that can cause intestinal or extra-intestinal diseases, including diarrhea, respiratory tract infections, wound infections, and septicemia, in humans and several animal hosts [65, 66]. *E. coli* and *C. albicans* often co-exist in human tissues and body fluids. Considering that they are common symbiotic bacteria found in the mammalian gut, their interaction deserves further investigation. Past studies have demonstrated that the interaction between *E. coli* and *C. albicans* is synergistic and that their combination significantly increases the risk of mortality when compared to that of either of them individually [67, 68]. The specific mechanism underlying the increase in mortality may be associated with the regulation of biofilm formation, biofilm dispersion, hyphae growth, and antifungal sensitivity of *C. albicans* by *E. coli* [67, 69]. Past reports have suggested that, after the formation of fungal/bacterial mixed biofilm, the formation of *C. albicans* biofilm increased by 2.2 times [67, 69] and the spread of *C. albicans* increased by 2.7 times. Moreover, the sensitivity of *C. albicans* to nystatin decreased, and the minimum inhibitory concentration increased from 25 µg/mL to 50 µg/mL [70]. ( Fig. 1A).

However, studies on the influence of the supernatant of *E. coli* biofilm on the development of *C. albicans* biofilm demonstrated that *E. coli* biofilm secretions could regulate *C. albicans* hypha-specific genes (*HSGs*) expression and significantly impair its biofilm development. This secretion considerably restrained *C. krusei*, *C. tropicalis*, *C. glabrata*, and *C. albicans* biofilms at 24 h, and all *Candida* spp. at 48 h [71]. (Fig. 1B) In addition, Cabral et al. reported that a soluble factor secreted by *E. coli* can kill *C. albicans* in a magnesium-dependent manner [72]..

### *C. albicans* and *Pseudomonas aeruginosa*

*P. aeruginosa*, a common gram-negative bacterium, is an important pathogenic factor that causes serious infections in humans. Owing to its natural resistance to antibiotics, an infection caused by this pathogen can result in serious clinical complications [73]. Clinically, *C. albicans* and *P. aeruginosa* are frequently isolated from various sites of the body and body fluids simultaneously, including the urine, venous ducts, the lungs of cystic fibrosis patients, and the gut [74]..

In patients with cystic fibrosis and ventilator-acquired pneumonia (VAP), the co-presence of *P. aeruginosa* and *C. albicans* is associated with a higher fatality rate [75, 76]. Colonization at the respiratory tract by *C. albicans* increases patients’ risk to develop a *P. aeruginosa* VAP [75, 77, 78]. Antifungal therapy for patients with *C. albicans* colonization in the tracheobronchus can reduce the risk of *P. aeruginosa* VAP or its colonization in the tracheobronchus [79], which, in turn, proves that *C. albicans* plays an important role in promoting *P. aeruginosa* infection. *C. albicans* has also been found to induce the growth of *P. aeruginosa* in the gut [80]..

Nevertheless, the two organisms have demonstrated obvious antagonism in a bi-species environment [81]. *P. aeruginosa* can produce a variety of phenazines that are harmful to *C. albicans*, such as pyocyanin (PYO) and 5-methyl-phenazine-1-carboxylic acid (5MPCA) [82, 83]. Past researchers used the analog PMS of 5MPCA to characterize the specific antifungal machinery of 5MPCA and found that phenazines could covalently bind soluble proteins in *C. albicans* biofilms in vivo. As a result, these soluble proteins of *C. albicans* were reduced by NADH and then spontaneously oxidized by oxygen to produce reactive oxygen species (ROS) [82]. Thus, the pathway of oxygen acquisition and respiration metabolism of *C. albicans* biofilm were blocked. Hence, the hyphal formation, intercellular adhesion, and biofilm development of *C. albicans* were inhibited [83]. In addition, cell–cell signaling molecules such as 3-oxo-C12 homoserine lactone produced by *P. aeruginosa* can hinder the filamentation of *C. albicans* [84]..

**Fig. 2:**
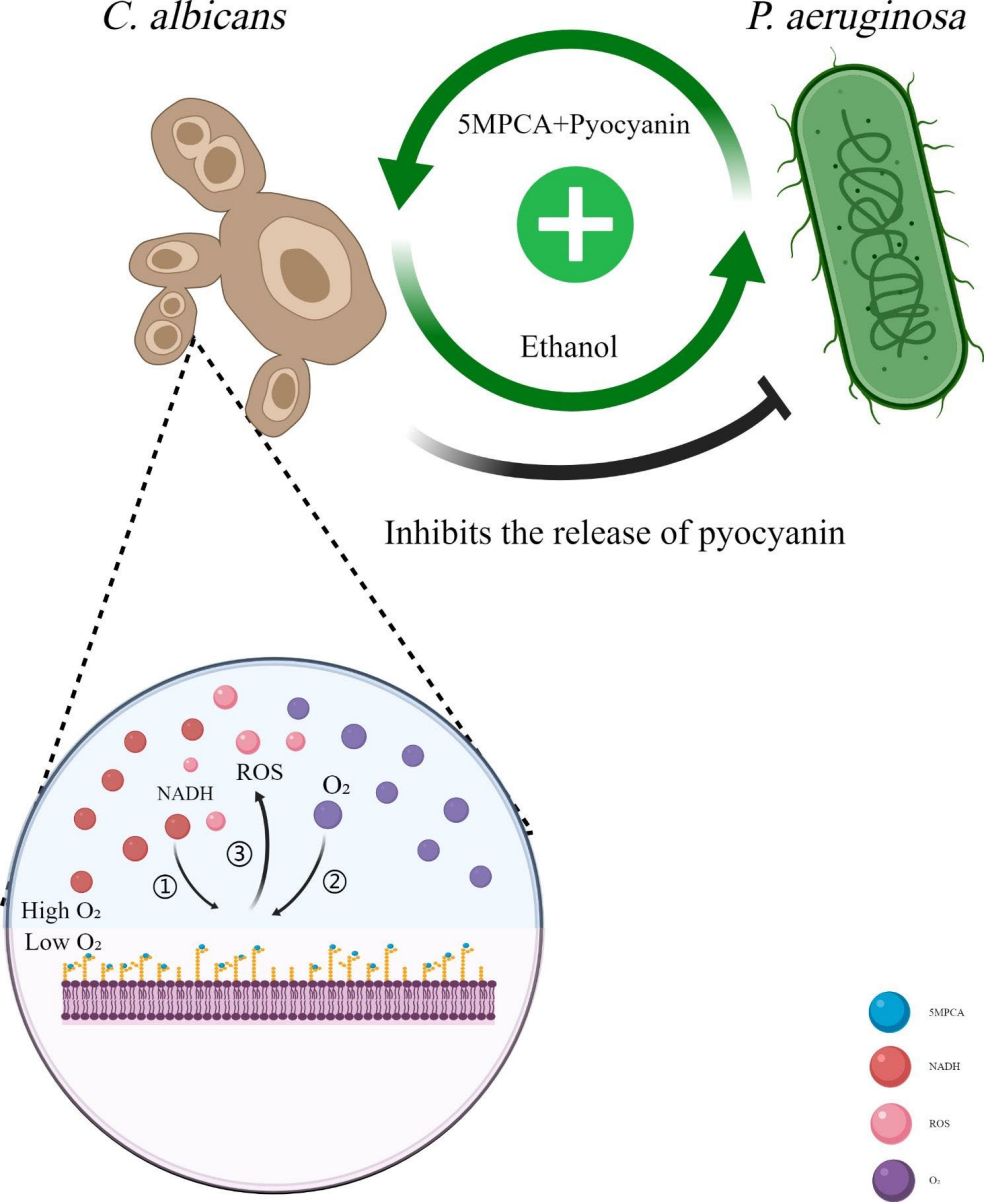
The interaction between *C. albicans* and *P. aeruginosa*. *P. aeruginosa* can secrete phenazines such as 5MPCA and pyocyanin to block the pathway of oxygen acquisition and respiration metabolism of *C. albicans*. Ethanol produced by *C. albicans* induced by phenazines can promote *P. aeruginosa* to secrete more phenazines. But *C. albicans* can reduce *P. aeruginosa*’ s virulence by inhibiting its release of pyochelin

Interestingly, ethanol produced by *C. albicans* induced by phenazines can promote *P. aeruginosa* to convert PCA into more phenazine final products such as PYO, phenazine-1-carboxamide, and 5MPCA. This positive feedback loop consisting of ethanol and phenazines drives a more *P. aeruginosa*-conducive interaction pattern between the two microbes [85]. However, for self-protection, *C. albicans* can reduce *P. aeruginosa*’ s virulence by inhibiting its release of pyochelin [86]. (Fig. 2).

### *C. albicans* and *Acinetobacter baumannii*

**Fig. 3:**
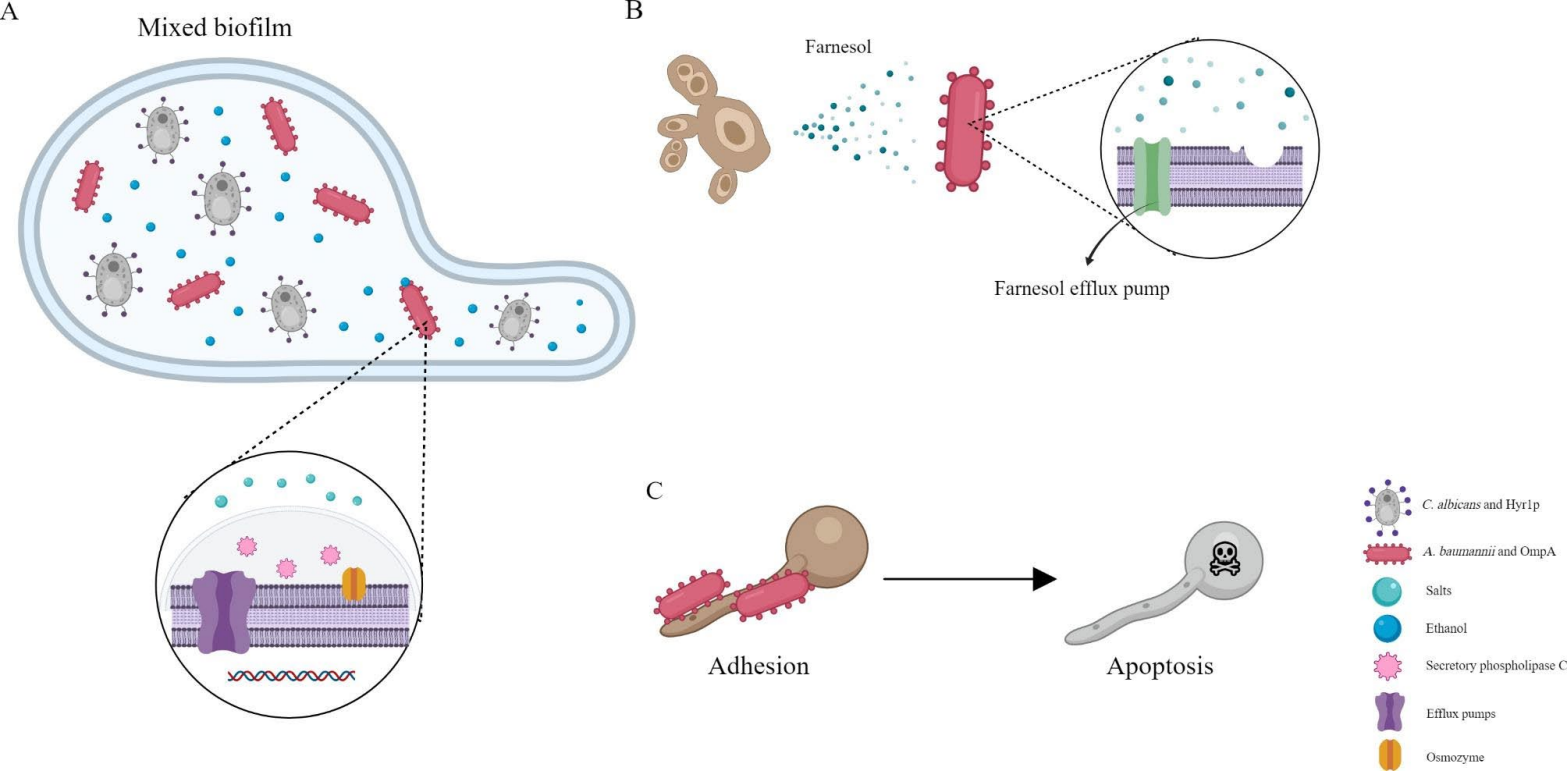
The interaction between *C. albicans* and *A. baumannii*. **(A)***C. albicans* and *A. baumannii* can utilize the homology of Hyr1p and OmpA to form a mixed biofilm. At the same time, ethanol secreted by *C. albicans* can upregulate a range of genes in *A. baumannii*, making it more likely to survive. **(B)** Farnesol secreted by *C. albicans* can disrupt the membrane integrity of *A. baumannii*, but *A. baumannii* can use an efflux pump against it. **(C)***A. baumannii* can attenuate the virulence of *C. albicans* by attaching the OmpA to *C. albicans* hyphae in order to induce its apoptosis

*A. baumannii* is certainly a very dangerous germ that can cause hospital-acquired infections (HAI) in the current healthcare systems, often causing refractory periodontitis, ventilator-associated infections and blood infections in critically ill patients. Owing to its multidrug-resistant nature, only rare antibiotics can cure infection caused by *A. baumannii*. Hence, the transmission of multidrug-resistant *A. baumannii* is worrying [87–89]. The gut, which serves as the body’s main reservoir for *A. baumannii*, may play an important role in the multidrug resistance of *A. baumannii* [90], which also arouses our curiosity about its interaction with *C. albicans*. Respiratory colonization of *C. albicans* has been reported to be an independent risk factor for *A. baumannii* VAP [91]. In a rat model of respiratory colonization of *C. albicans*, researchers found that the colonization of *C. albicans* made rats more prone to *A. baumannii*-associated pneumonia, with a higher CFU burden of *A. baumannii* and more severe lung damage [92]. The reason why *C. albicans* can make *A. baumannii* more likely to survive that, as a fungus, the ethanol produced by *C. albicans* can not only serve as a carbon source for *A. baumannii* but also upregulate the expression of 49 genes in *A. baumannii*, including genes encoding efflux pumps, secretory phospholipase C, osmozyme, and iron assimilation. In addition, ethanol can induce the high-affinity phosphate transport system of *A. baumannii* and help resist the toxic effects of salts [93, 94]. In addition, because of the significant structural homology between *A. baumannii* outer membrane protein A (OmpA) and *C. albicans* hyphal wall protein Hyr1p, Hyr1p can be used as a receptor for the binding of *A. baumannii* and *C. albicans* to form mixed-species biofilms [95]. ( Fig. 3A).

However, the interaction between *C. albicans* and *A. baumannii* is also paradoxical. It was found that the secretion of farnesol by *C. albicans* disrupts the membrane integrity of *A. baumannii*, impairs its virulence characteristics, and alters its cell morphology. However, *A. baumannii* can use an efflux pump against farnesol, which may work as a defense mechanism [96]. (Fig. 3B) In addition, *A. baumannii* can attenuate the virulence of *C. albicans* by inhibiting its filamentation process and attaching the outer membrane protein A (OmpA) to *C. albicans* hyphae in order to induce its apoptosis [97–99]. (Fig. 3C).

### *C. albicans* and *Enterococcus faecalis*

**Fig. 4:**
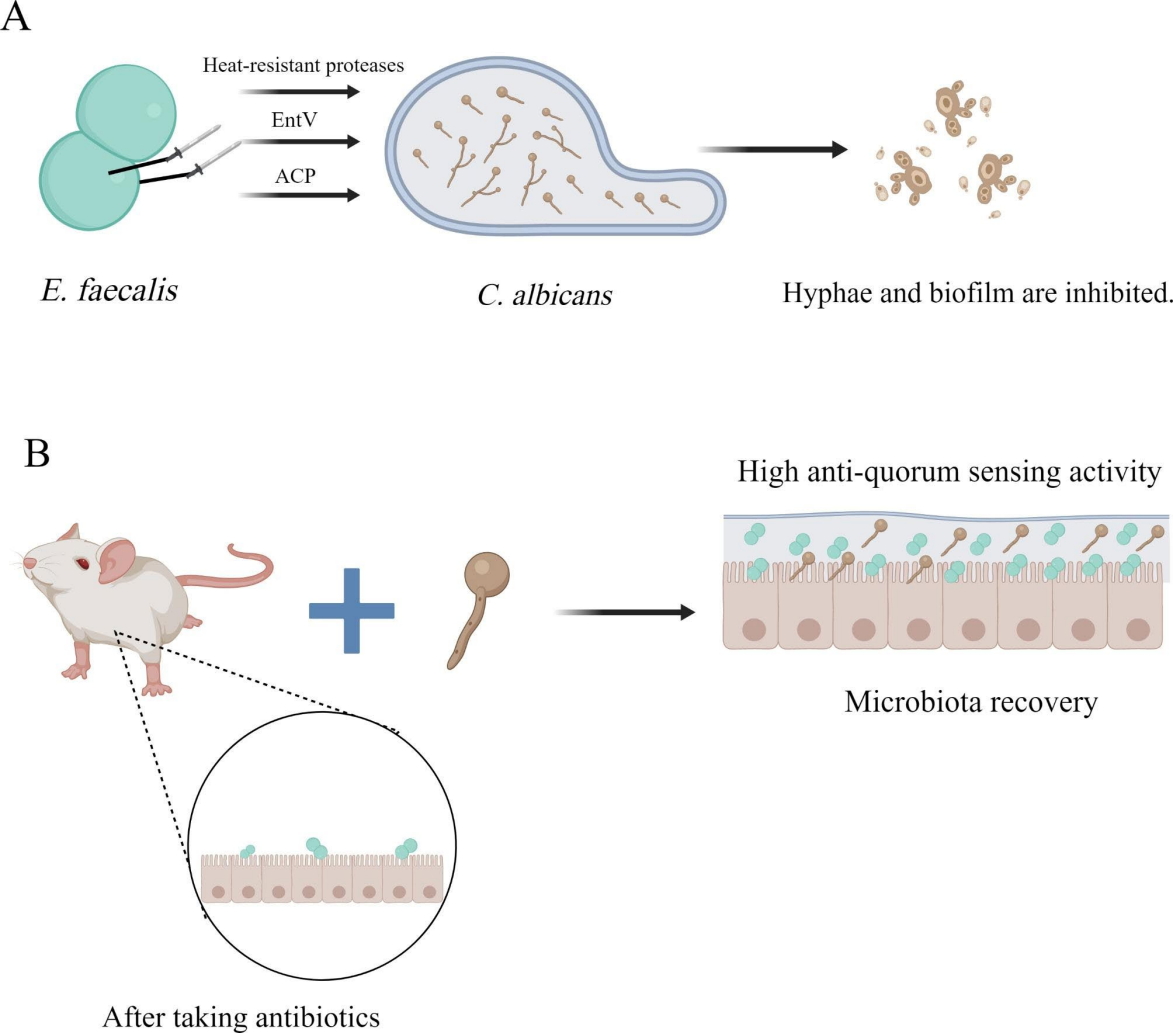
The interaction between *C. albicans* and *E. faecalis*. **(A)***E. faecalis* can secrete EntV, ACP and heat-resistant proteases to block the biofilm development of *C. albicans* and inhibit its virulence. **(B)***C. albicans* can help *E. faecalis* recover in antibiotic-treated gut

*E. faecalis* is a gram-positive pathogen. It is ubiquitous and can survive in various natural environments, including the human body. As an opportunistic pathogen, it colonizes the human gut surface, forms a biofilm, contributes to severe hospital infections, and shows high resistance to several antibiotics [100, 101]. Past studies have reported that *E. faecalis* has a protective effect against *C. albicans* infection. After oral administration of heat-inactivated *E. faecalis* to mice infected with *C. albicans*, *E. faecalis* can prevent thrush in mice by interacting directly with *C. albicans* in vivo and stimulating the host to enhance the immune response [102]. In a multi-microbial model of *C. elegans*, *E. faecalis* was observed to secrete heat-resistant proteases GelE and SerEin by relying on the Fsr quorum-sensing system to inhibit the hyphae morphogenesis of *C. albicans*, thereby negatively affecting its virulence [103]. *E. faecalis* can also encode EntV to block the biofilm development of *C. albicans* on a solid matrix and disrupt its pre-formed biofilm against the current antifungal drugs. The peptide also protects macrophages and enhances their antifungal abilities. These results suggest that EntV may be used as a potential fungal agent against *C. albicans* in the future [104]. Furthermore, Shekh et al. isolated and purified a non-hemolytic anti-*C. albicans* protein (ACP) from *E. faecalis* for the first time and proposed that this protein could be used to treat candidiasis in the future [105]. (Fig. 4A).

Although several studies have demonstrated that *E. faecalis* could help in the treatment of *C. albicans* infection, a study found that when *E. faecalis* and *C. albicans* were co-infected, a thicker, denser biofilm with stronger tolerance to harmful stresses was formed on root canal dentin and glass slides, which can increase bone resorption of osteoclasts, inhibit the bone formation of osteoblasts, upregulate inflammatory cytokines such as IL-6 and TNF-α, and ultimately increase the severity of dental pulp diseases. Moreover, past studies have reported that *C. albicans* can help *E. faecalis* become more resistant to starvation [106, 107]. Past studies using mouse models also demonstrated that the existence of *C. albicans* in cefoperazone-treated gut facilitated the rehabilitation of *E. faecalis* during antibiotic recovery [108, 109]. Meanwhile, the metabolites of carbohydrates, amino acids and polyamines in the mixed biofilm of the two changed, displaying higher anti-quorum sensing (QS) activity compared to that of a single biofilm [110]. (Fig. 4B).

### *C. albicans* and *Staphylococcus aureus*

*S. aureus* is a clinically important pathogen that can give rise to various infections in the body, including mild skin infections, severe tissue infections, and septicemia, and it is a common reason for hospital-acquired and community-acquired infections [111]. Because *S. aureus* and *C. albicans* share several common host niches, including the gut, both are often co-isolated from mixed fungal-bacterial infections. Several studies and clinical cases have demonstrated that *S. aureus* and *C. albicans* have an infectious synergistic effect. The co-inoculation of *C. albicans* and *S. aureus* can cause more severe and extensive infection and higher mortality than the inoculation of either species alone [112–114]. Signaling pathways controlled by Efg1, a transcription factor used to induce *C. albicans* hyphal gene expression and hyphae growth, have been reported to be critical for *C. albicans* to strengthen *S. aureus*’s virulence in abdominal infection [115, 116]. However, *C. albicans*’ s ability to enhance *S. aureus*’s virulence in the peritoneal cavity has not been correlated with the presence or absence of the hyphae of *C. albicans*, suggesting that when the two microorganisms co-infect the peritoneal cavity, there may be other processes independent of morphology that are regulated by Efg1, which result in fatal synergies [117, 118].

**Fig. 5:**
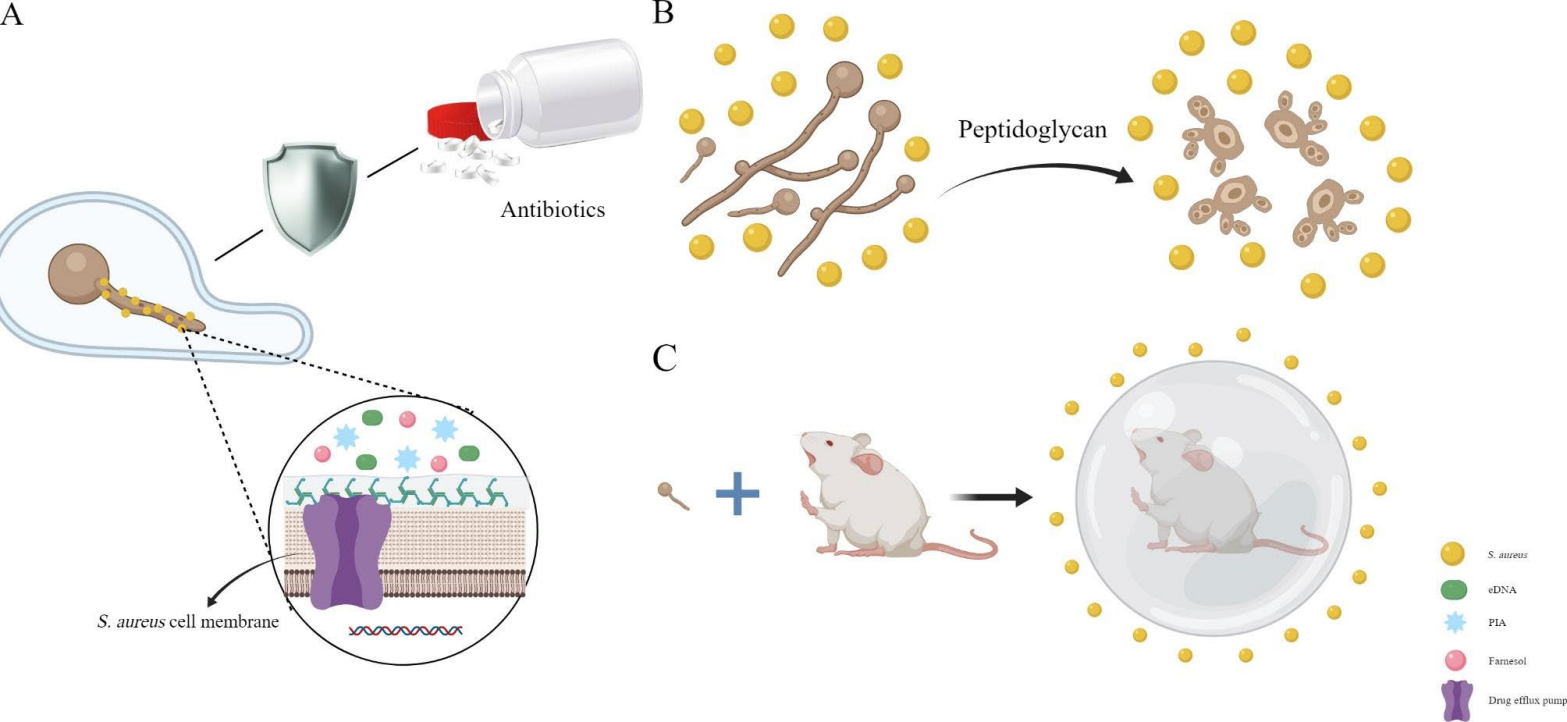
The interaction between *C. albicans* and *S. aureus*. **(A)***C. albicans* can upregulate a range of genes that promote *C. albicans* to express drug efflux pumps and small molecules that help form network structures in biofilms to resist antibiotics. (**B)***S. aureus* can secrete peptidoglycans to affect the metabolism, morphogenesis, and virulence of *C. albicans*. **(C)** Inoculating separately can induce a protective effect on the host, possibly because of the trained immunity induced by β-glucan of *C. albicans*

In addition, co-infection with *C. albicans* and *S. aureus* reduces the sensitivity of *S. aureus* to antibiotics [113, 119]. Vancomycin has been reported to have a significant effect on the formation of a single biofilm of *S. aureus*, but a significantly reduced effect on mixed biofilms [119]. This effect is related to the adhesion between the two, farnesol-induced upregulation of the *S. aureus* drug efflux pump and increased eDNA and polysaccharide intercellular adhesin (PIA) in *S. aureus* biofilms, which induce the formation of network structures [120]. (Fig. 5A).

Interestingly, virulence factors and superantigen-like proteins, which play a huge role in the spread of *C. albicans* infection, were downregulated during co-infection, whereas the capsular polysaccharide gene was upregulated during co-infection, suggesting that *C. albicans* adopts a strategy that is more conducive to survival, as well as increased persistence and immune evasion of the host in cases of mixed infections [119]. The specific mechanism behind this may be that *S. aureus* affects the metabolism, morphogenesis, and virulence of *C. albicans* by producing peptidoglycans [117]. (Fig. 5B).

However, a protective effect on the host can be induced when the timing of *C. albicans* and *S. aureus* inoculation is different, possibly because of the trained immunity induced by β-glucan of *C. albicans*, which provides cross-protection against secondary *S. aureus* infection [121–126]. (Fig. 5C).

### *C. albicans* and *Salmonella*

**Fig. 6:**
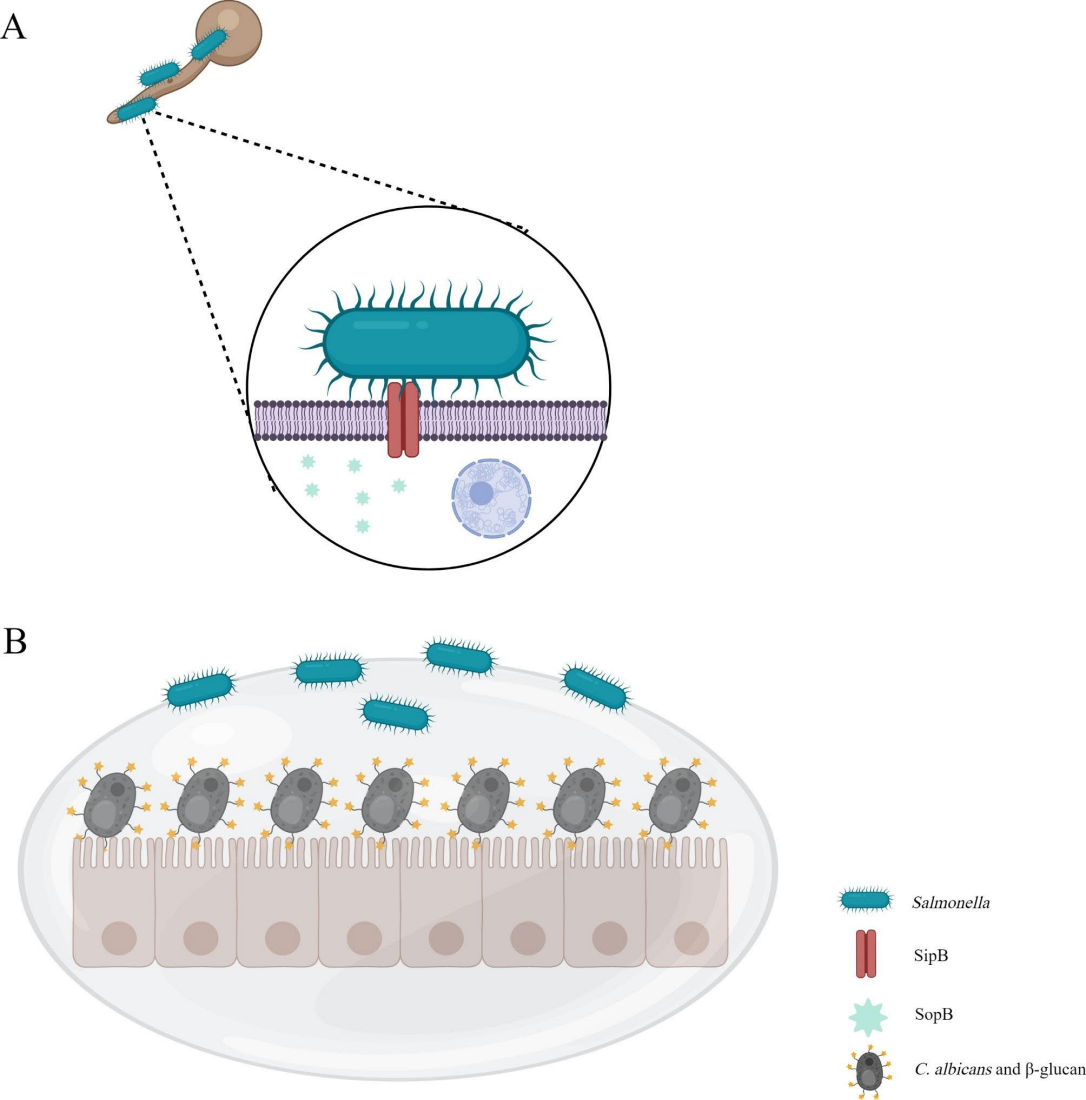
The interaction between *C. albicans* and *Salmonella*. **(A)***Salmonella* can inject SopB into *C. albicans* through SipB to attenuate its virulence. **(B)***C. albicans* can use β-glucan to inhibit the colonization of *S. typhimurium* in the gut

*Salmonella* is a gram-negative bacterium that causes gastrointestinal lesions ranging from asymptomatic carriers to gastroenteritis and typhoid fever [127, 128]. Based on the current studies, the relationship between *Salmonella* and *C. albicans* is only antagonistic. Past studies have demonstrated that when *C. elegans* are infected with both *C. albicans* and *Salmonella typhimurium*, the filamentation of *C. albicans* is hindered [129]. The underlying action mechanism is that the type III secretion systems encoded by SPI-1 and SPI-2 by *Salmonella* genes directly inject various effector proteins into *C. albicans* to exert the virulence of this species [130–132]. Among these effector proteins, the one encoded by *sopB* can be translocated into *C. albicans* hyphae through SipB to downregulates the transcription of *CDC42*, thereby destroying *C. albicans’ s* hyphae to attenuate its virulence [133]. (Fig. 6A).

Correspondingly, *C. albicans* can use β-glucan, an immunomodulatory substance in its cell wall, to inhibit the colonization of *S. typhimurium* in the gut and liver [134]. (Fig. 6B).

### *C. albicans* and *Helicobacter pylori*

*H. pylori*, a gram-negative microaerobic bacterium, grows in the human gastroduodenal mucosa, causes inflammation and gastrointestinal diseases, and increases the risk of gastric cancer development [135, 136]. As a facultative intracellular bacterium, *H. pylori* can parasitize human gastric epithelial cells and immune cells. It has evolved to use the vacuoles of eukaryotic cells as a protective niche, which can help avoid the harsh gastric environment and produce obvious resistance to antibiotics, thereby allowing reproduction and persistence in the host for a long time [137–141]. *H. pylori* has been found to move and survive in the vacuole of *C. albicans*, suggesting that *C. albicans* can be used as a host and carrier by *H. pylori* to provide an alternative niche [142]. This is an unusual evolutionary phenomenon because the fungal cell wall typically limits endocytosis and the uptake of bacteria [143]. In this process, *H. pylori* can fuse its *vacA* s1s2, *ureAB*, *16 S rRNA*, and *ahpC* genes into *C. albicans* DNA and create an ideal shelter for itself by taking advantage of the good tolerance of *C. albicans* to stress conditions, so that it can obtain nutrition, express proteins, and reproduce in *C. albicans* cells and continue to exist and reinfect the host [144–147].

**Fig. 7:**
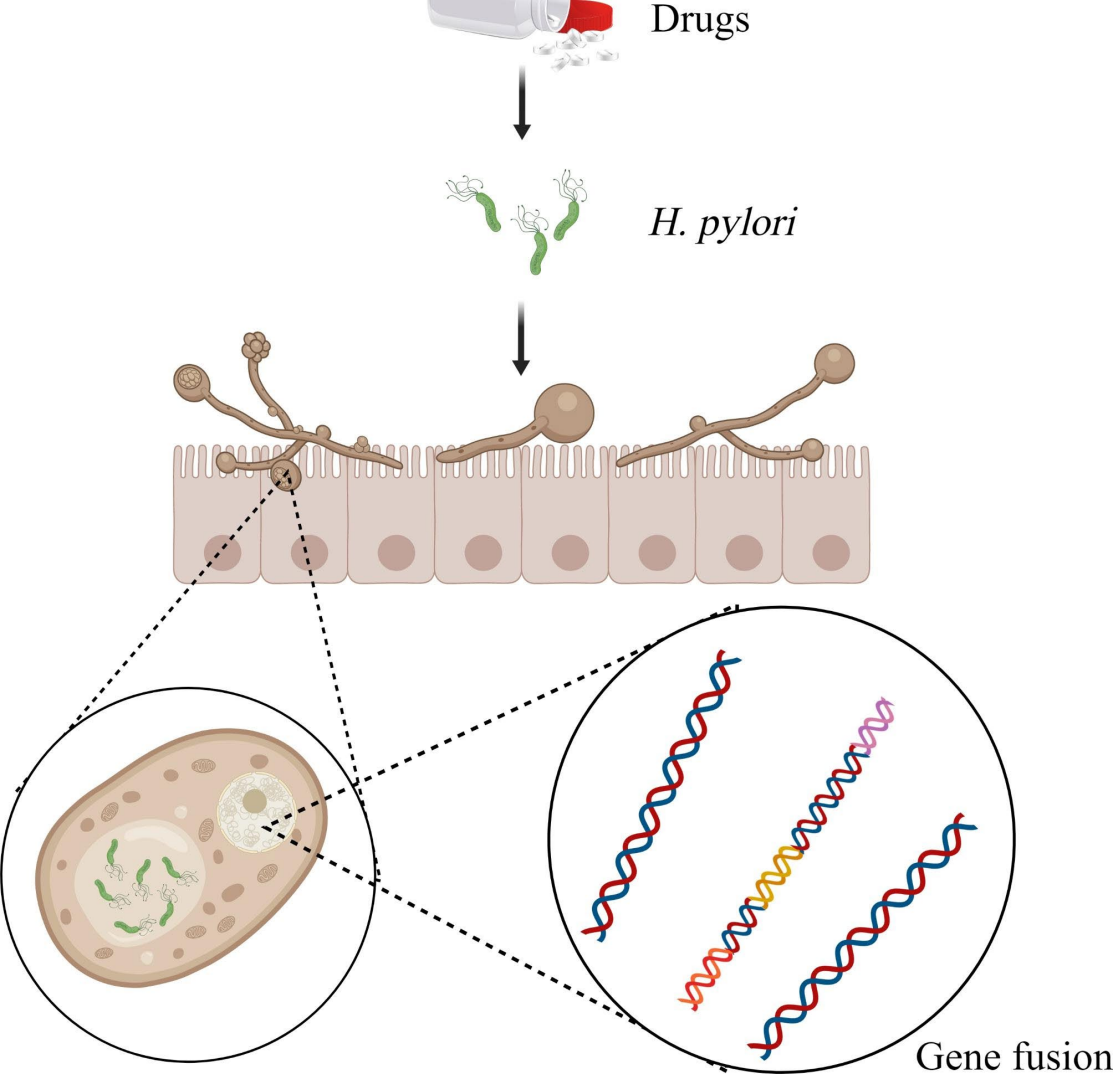
The interaction between *C. albicans* and *H. pylori*. *H. pylori* can be parasitic in *C. albicans*, especially under the pressure of drugs

Stress from the physicochemical environment and drugs in the gut is a powerful booster for *H. pylori* to enter *C. albicans*. A previous study showed that *H. pylori*, which can only adapt to fluctuations in pH in the range of 6–8, entered the cell of *C. albicans*, which can accept fluctuations in pH in the range of 2–10, to protect itself in an acidic environment that is not conducive to its survival; this phenomenon is more evident when the pH is lower [148]. Similarly, when *H. pylori* are treated with antibiotics such as amoxicillin, more numbers of *H. pylori* cells can be observed to enter *C. albicans* cells than usual, which causes treatment failure to a large extent [149]. (Fig. 7)Meantime, when *H. pylori* patients are older and their proton pump inhibitors (PPI) intake is higher, *C. albicans*’ s colonization in the human stomach gains an advantage, thus forming a positive cycle of mutual coordination [150]. On researching how *H. pylori* are safely released from *C. albicans*, some studies found that *H. pylori* can be released from *C. albicans* in the form of vesicles or free bacteria without causing damage to *C. albicans* [151]..

This combination of pathogenic microorganisms is undoubtedly a great threat to human health, and oral and fecal-oral transmission of *C. albicans* also contributes to the wider spread of *H. pylori*. Moreover, *C. albicans* in the vagina is more efficient to *H. pylori* colonization than *C. albicans* in the mouth. If *C. albicans* is transmitted from the mother’s vagina to the mouth of the newborn, it may significantly increase the risk of *H. pylori* infection in the newborn [152]..

However, the relationship between *H. pylori* and *C. albicans* is not entirely mutually beneficial. Some studies have shown that the peptide HP [2–20] produced by *H. pylori* is highly toxic to *C. albicans* [153]. HP [2–20] can destroy the cell membrane structure of *C. albicans* or directly interact with the lipid bilayer, thereby increasing the outflow of potassium ions, reducing the intracellular trehalose content of *C. albicans*, and eventually exerting antifungal activities [153–156].

### *C. albicans* and *Lactobacillus*

**Fig. 8:**
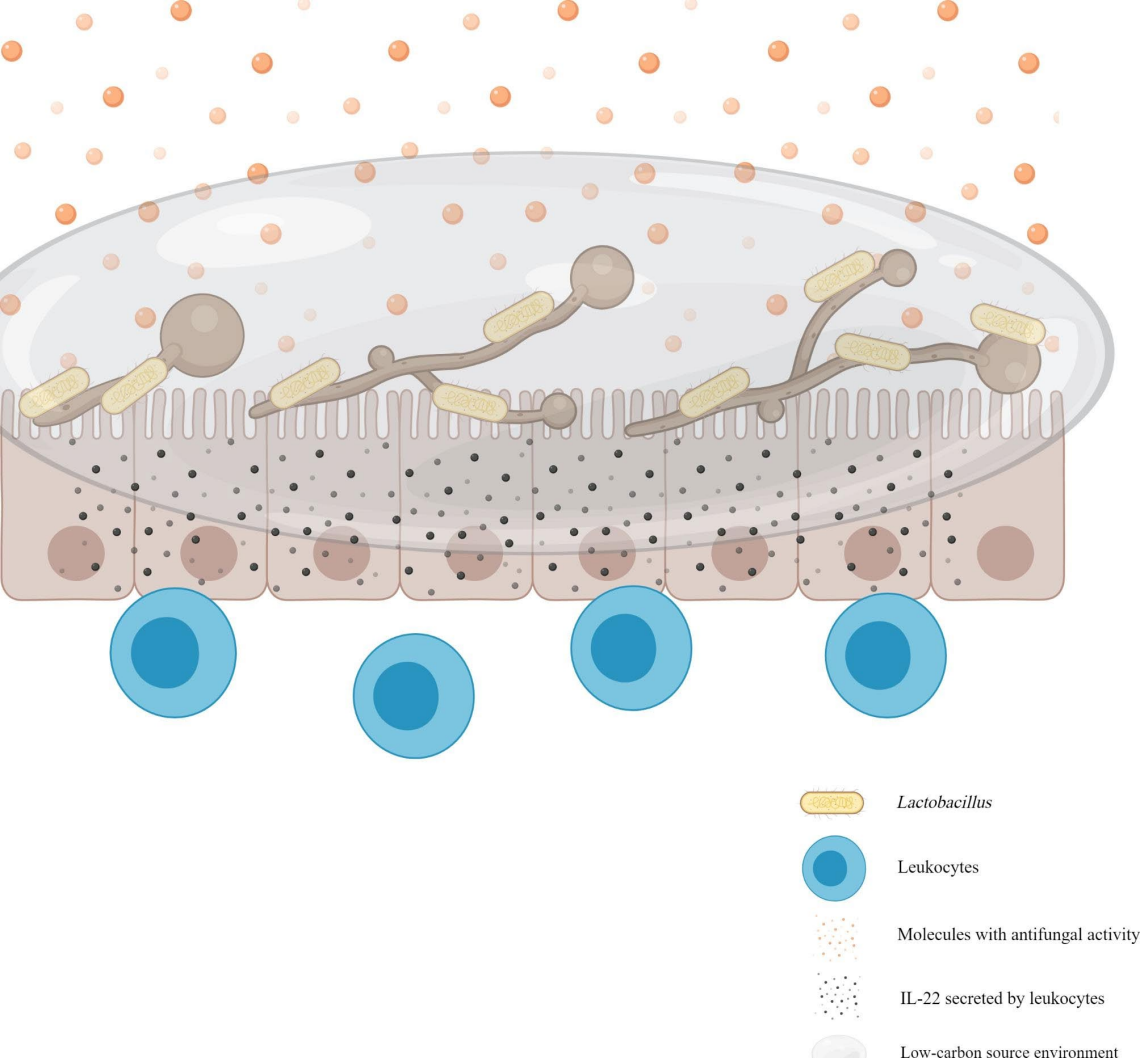
The interaction between *C. albicans* and *Lactobacillus*. *Lactobacillus* can antagonize *C. albicans* by physical adhesion, secretion of molecules with antifungal activities, reshaping the metabolic environment and activating leukocytes in the gut

The genus *Lactobacillus* is taxonomically complex and consists of > 170 gram-positive species. Although they are a part of the normal human gastrointestinal and vaginal flora, they may act as opportunistic human pathogens [157]. They are often widely used in the preparation of various commercial products, as well as probiotics [158]. Several studies have demonstrated an obvious antagonism between *Lactobacillus* and *C. albicans*. *Lactobacillus* can protect against intestinal epithelial necrotizing injury caused by *C. albicans* [159, 160]; the supplementation of *Lactobacillus* and *Bifidobacterium* in premature and low-birth-weight infants can reduce *C. albicans* colonization in the gastrointestinal tract, thereby reducing the incidence of *C. albicans* sepsis and infant mortality [161]. Past studies have reported that the protection was time- and dose-dependent and independent of competition for the adhesion sites. This mechanism can be classified as direct physical antagonism and chemical antagonism with soluble molecules [25, 159]. In physical antagonism, *Lactobacillus* directly interacts with *C. albicans* and causes it to detach from the gut mucosa [159]. Chemical antagonism can be categorized as reshaping the metabolic environment of *C. albicans* (e.g., it consumes the main nutrient source of *C. albicans* and forces it to change its metabolic mode, thereby weakening its toxicity) and as a direct secretion of molecules with antifungal activities (e.g., lactic acid, short-chain fatty acids, hydrogen peroxide, bacteriocin-like substances, and biosurfactants) [25, 159, 162, 163]. In addition, *Lactobacillus* can produce indole-3-aldehyde, which acts on aryl hydrocarbon receptor (AhR) and activates leukocytes. The activation of group 3 ILCs (ILC3s) and regulatory T (Treg) cells produce a large amount of IL-22, which can hinder the colonization of *C. albicans* in the gut mucosa and create gut mucosal homeostasis that allows the survival of mixed microbial communities [164]. (Fig. 8) Moreover, *Lactobacillus* can inhibit the expression of drug efflux protein produced by drug-resistant *C. albicans* and reverse its drug resistance [165]..

Under an appropriate conditions, *C. albicans* launches a counterattack against *Lactobacillus*. Past studies have shown that *C. albicans* prevented *Lactobacillus* regrowth in the stomach of mice treated with cefoperazone, an effect that persisted for at least 3 weeks after antibiotic treatment was discontinued, which induced stomach inflammation. However, this phenomenon can be easily suppressed. Some studies have shown that restoring the bacterial community in the stomach of mice within a week of discontinuing antibiotics was adequate to inhibit the development of gastritis [108, 109, 166].

### *C. albicans* and *Bacteroides*

**Fig. 9:**
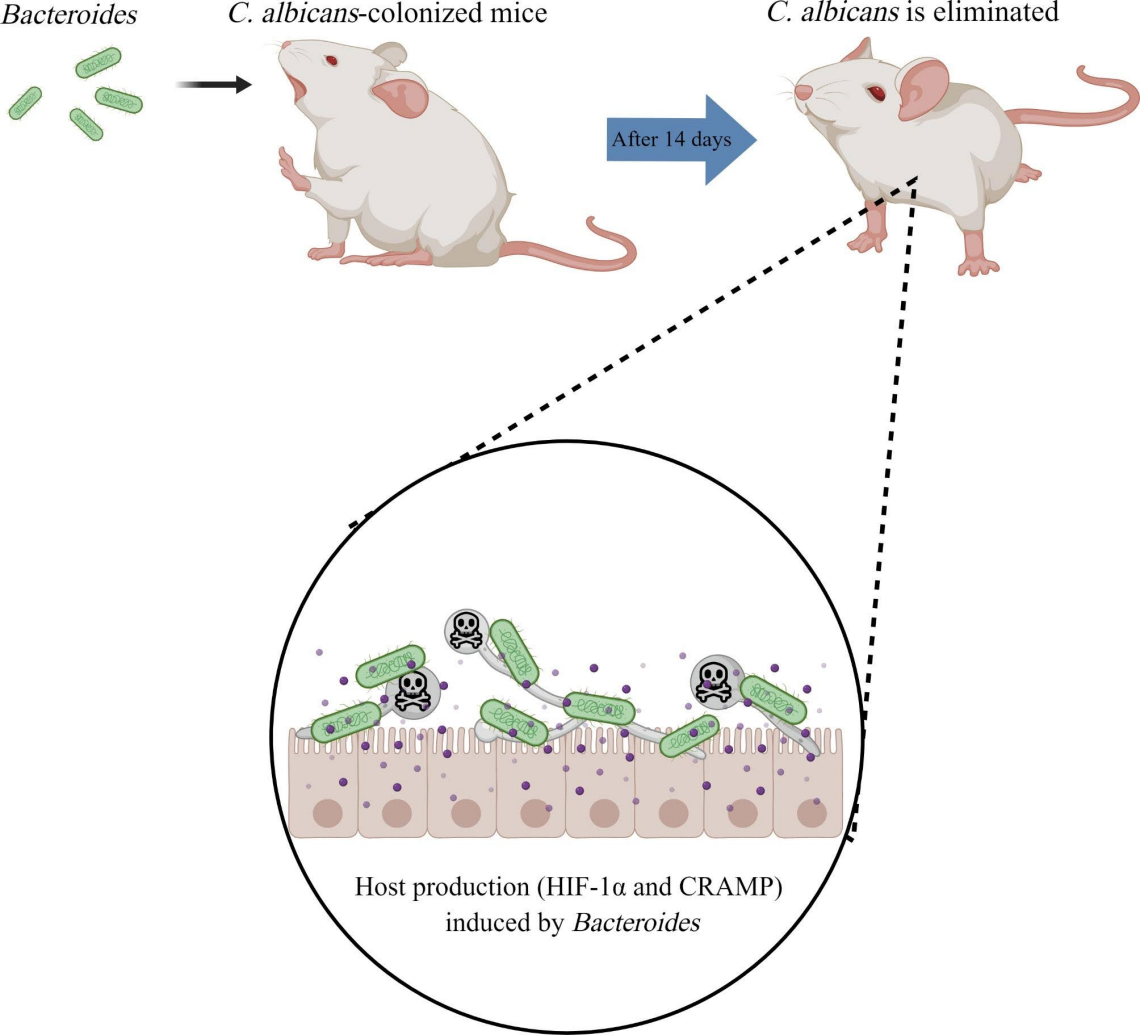
The interaction between *C. albicans* and *Bacteroides*. *Bacteroides* can induce host production to promote the eliminated of *C. albicans* from the gut in antibiotic-treated *C. albicans*-colonized mice

*Bacteroides* are clinically important pathogens that are commonly associated with most anaerobic infections, with a death rate of > 19% [167]. *Bacteroides* maintain a complex and beneficial relationship with their host when they remain in the gut, and their leaving this environment can induce significant pathological changes such as bacteremia and multiple abscesses all over the body [167]. Studies have found that *Bacteroides* can hinder *C. albicans*’s growth and virulence, making them the most effective bacterial group to promote the colonization resistance of *C. albicans* [168]. The oral administration of *Bacteroides fragilis* to antibiotic-treated *C. albicans*-colonized mice showed that *C. albicans* was eliminated from the gut after 14 days. During this process, host production (HIF-1α and CRAMP) induced by *Bacteroides* played a huge role in maintaining the colonization resistance of *C. albicans* [169]. As an important regulator of the mammalian innate defense, HIF-1α upregulates the expression of cathelicidin-related antimicrobial peptides (CRAMP) in the bone marrow cells, which play a key role in mammalian natural immune defense against bacterial infection. The human cathelicidin LL-37 has been demonstrated to possess anti-*C. albicans* ability to hinder *C. albicans* adhesion to the epithelium by preferentially binding to the components of *C. albicans* cell wall such as mannan, chitin, and dextran [170–173]. (Fig. 9).

Although these studies demonstrated that *Bacteroides* are almost lethal for *C. albicans*, the mucin produced by *Bacteroides thetaiotaomicron* has been found to promote the growth of *C. albicans* [174]. In addition, in the cecum of cefoperazone-treated mice, the existence of *C. albicans* facilitated the rehabilitation of *Bacteroides* during cefoperazone treatment [109, 166]. A possible mechanism for this is that *C. albicans* provides a hypoxic microenvironment for *Bacteroides* through aerobic respiration and produces antioxidants to promote its growth [175, 176].

### *C. albicans* and *Clostridium difficile*

*C. difficile*, an anaerobic toxin-producing bacterium, is the main reason behind hospital-acquired infections. Multiple surface proteins and flagella allow *C. difficile* to colonize the gut, where it can cause gut diseases ranging from mild diarrhea to deadly infectious colitis, leading to significant morbidity and mortality worldwide [177, 178]. Fecal microbiota transplant (FMT) treatment in *C. difficile* infections (CDI) model mice demonstrated that the existence of *C. albicans* reduced the efficacy of FMT, whereas antifungal therapy restored the efficacy, indicating that *C. albicans* promotes *C. difficile*’s survival [179]. The underlying mechanism behind this may be that the existence of *C. albicans* creates an anoxic microenvironment for *C. difficile*, which allows *C. difficile* to continue to grow under aerobic conditions [175, 180]. Meanwhile, higher *C. albicans* infection rates have been reported in CDI patients, suggesting that *C. difficile* may promote the colonization of *C. albicans* [179, 181].

However, similar to *Bacteroides* species, *C. difficile* is the key to maintaining resistance to the colonization of *C. albicans* in the mice gut [169]. Past studies have demonstrated that taurocholate acid (TCA) can promote the colonization and transmission of *C. albicans* in the gastrointestinal tract by significantly reducing the abundance of *C. difficile*, which indirectly indicates that *C. difficile* plays a certain antagonistic role in the gastrointestinal tract against *C. albicans* [182]. In addition to activating HIF-1α and inducing CRAMP expression similar to *Bacteroides* for reducing gut colonization and post-infection mortality associated with *C. albicans*, *C. difficile* can produce para-cresol, a bacteriostatic compound that can alter the morphology, inhibit biofilm formation, and antagonize the growth of *C. albicans* via tyrosine fermentation [169, 180, 183].

### *C. albicans* and *Streptococcus*

**Fig. 10:**
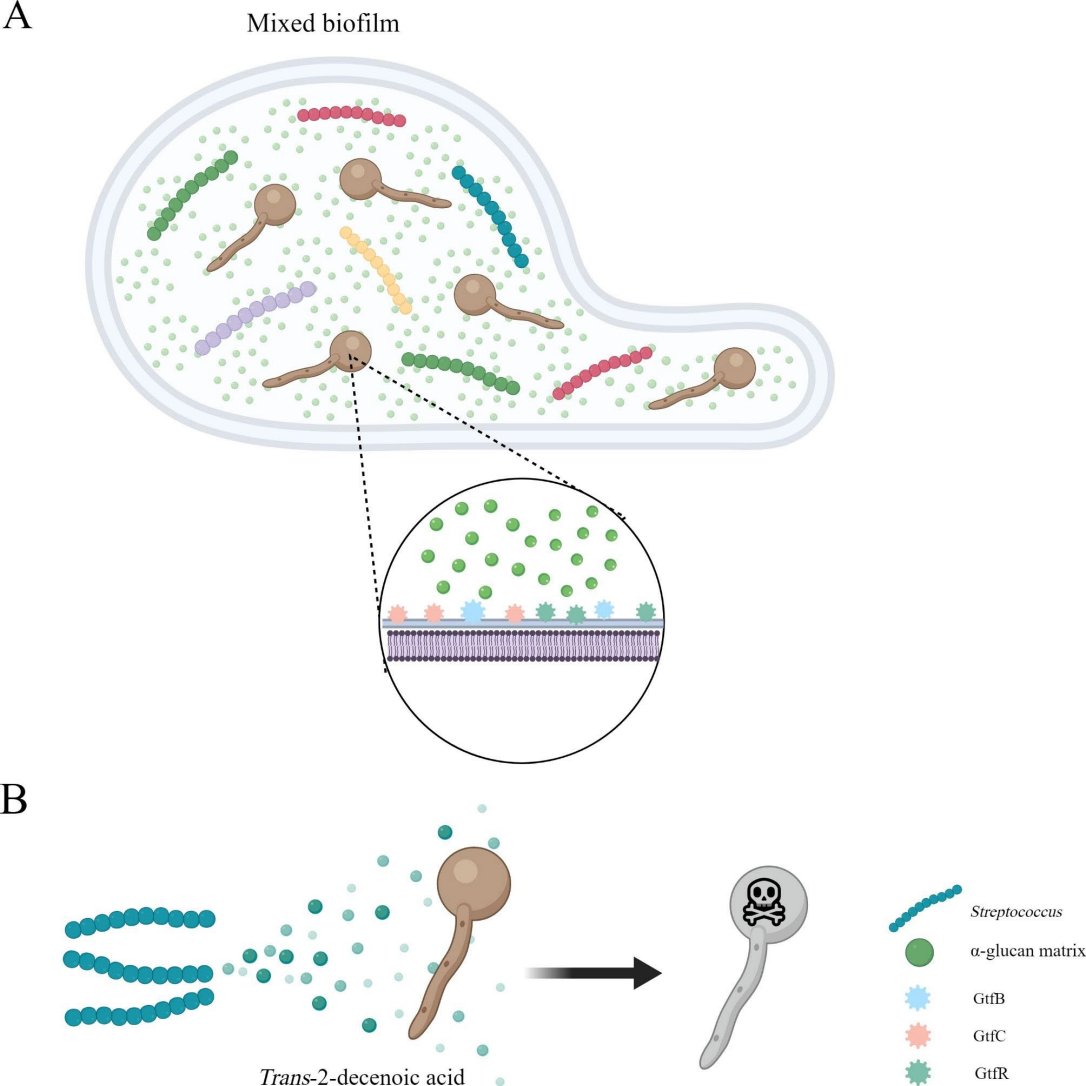
The interaction between *C. albicans* and *Streptococcus*. **(A)** After *C. albicans* and *Streptococcus* form a mixed biofilm, *Streptococcus* induces *C. albicans* to produce a large amount of α-glucan by secreting glucotransferases. **(B)***Trans*-2-decenoic acid secreted by *Streptococcus* inhibits *C. albicans*’s virulence

*Streptococcus* is a common gram-positive coccus, consisting of 104 strains, which mainly cause otitis media, pneumonia, bacteremia, and meningitis. When *Streptococcus* colonizes the digestive tract, it can cause diseases in the digestive system [184]. Because of their niche preference, *Streptococcus* and *C. albicans* mainly coexist in the human mouth. Presently, most studies on the interaction between *Streptococcus* and *C. albicans* are related to oral diseases. However, both microorganisms can be found and extracted from the gut as well. Because *Streptococcus* is composed of a complex variety of strains, the interactions and mechanisms between different *Streptococcus* types and *C. albicans* are different. Several *Streptococcus* strains have been confirmed to co-aggregate with *C. albicans* such as *S. sanguis*, *S. gordonii*, *S. mutans*, *S. oralis*, and *S. anginosus* [185]. Among them, *S. mutans* has been reported to form synergies with *C. albicans*. In early childhood caries, there occurs a cross-feeding mechanism between these two microorganisms, which is mediated by the glucotransferase GtfB secreted by *S. mutans*. GtfB can bind to mannan on the outer surface of the *C. albicans* cell wall to produce a large number of the α-glucan matrix into the mixed biofilm, which not only promotes the formation of mixed biofilms and provides enhanced binding sites for the two microorganisms but also improves the utilization of carbohydrates by *C. albicans*, thereby increasing the severity of the disease [186–189]. GtfC and GtfR play a role similar to that of GtfB in the interaction between the two microorganisms [190–192]. (Fig. 10 A) In addition, the antigen I/II of *S. mutans* can mediate the increase in the number of *C. albicans* and the production of acids in the mixed biofilm [193]. In the process of interaction, *C. albicans* promotes the growth of *S. mutans* by secreting farnesol and polysaccharides [188, 194]. In addition to *S. mutans*, another representative *Streptococcus* strain that can form a synergistic effect with *C. albicans* is *S. gordonii*, which promotes biofilm formation, filamentation, and adhesion of *C. albicans*. The formation of dual-species biofilm also shows a high degree of resistance to combined antifungal and antibacterial treatment [195–198]. The key to this interaction is the glucosyltransferase GtfG [199]. In addition, *C. albicans* can promote the growth and metabolism of *S. gordonii* by increasing the activity of *S. gordonii* cell-wall-anchored glycoside hydrolases [200]..

However, the interaction between *Streptococcus* strains and *C. albicans* is also not a single synergy. Several *Streptococcus* strains mentioned above can secrete *trans*-2-decenoic acid to terminate the expression of *HWP1* associated with the formation of *C. albicans* hyphae, thereby inhibiting its virulence [201]. (Fig. 10B)Another *Streptococcus*, *S. gordonii*, has been found to secrete competence-stimulating-peptide (CSP), which hinders *C. albicans*’s biofilm-formation [201]..

## Conclusions

The colonization of *C. albicans* in the gut is a complex process that requires suppression of its filamentation and transformation into other cell types. In addition, C. albicans is required to adapt to the metabolic environment of the gut, resolve the carbon source problem as well as the intake and discharge balance of other micronutrients. Under the intestinal physical and chemical environmental pressures, the responses of C. albicans created by various signal pathways can determine its capacity to colonize the gut.

After colonizing the gut, the interactions between *C. albicans* and the gut bacteria become a significant part of the host’s health. With the continuous emergence of new findings, our understanding of the interactions between *C. albicans* and various gut bacteria continues to deepen. In general, the nature of the interactions of microbes can be classified into 5 broad categories: direct physical contact, chemical interaction by small secreting molecules involved in quorum sensing, alterations in host’s immune response, competition for carbon sources, and parasitism.

Among all the bacteria studied in this review, *P. aeruginosa*, *Lactobacillus spp.* and *Salmonella spp.* exhibit only antagonism against *C. albicans*, indicating that they are promising as the key to the treatment of *C. albicans* in the future [202]. Meanwhile, for bacteria that can secrete small molecules that antagonize *C. albicans*, purifying these secretions (e.g., soluble factors secreted by *E. coli*; phenazines and 3-oxo-C12 homoserine lactone secreted by *P. aeruginosa*; OmpA of *A. baumannii*; GelE, SerEin, EntV, and ACP secreted by *E. faecalis*; HP protein secreted by *H. pylori*; para-cresol secreted by *C. difficile*; *trans*-2-decenoic acid and CSP secreted by *Streptococcus spp.*) and using them to antagonize *C. albicans* is also a very promising approach. In addition, we can selectively knock out the synergistic genes of bacteria and introduce the mutant strains into the body, such as by knocking out the genes expressing various glucosyltransferases in *Streptococcus spp.* and only using its antagonism to *C. albicans*.

Nevertheless, several difficulties are encountered in the implementation of these treatment methods, such as the requirements of technical expertise in purifying small molecules, the dosage of small purified molecules to treatment, whether the harmful genes among the opportunistic pathogenic bacteria can be completely knocked out, and whether knocking out these bacterial genes have potential risks implications to the host health. All these aspects need to be constantly explored in practice. The synergistic effect of *C. albicans* and bacteria reveals that it is important to treat bacterial infections while treating *C. albicans* infection. We can apply their synergy to the treatment of mixed microbial infections. For example, owing to the homologous nature of Hyr1p of *C. albicans* and OmpA of *A. baumannii*, active immunization using rHyr1p-N or passive immunization using polyclonal antibodies against specific peptide motifs of rHyr1p-N provides new ideas for the future treatment for both *C. albicans* and *A. baumannii* [95]; Treatment of *C. albicans* is very beneficial in the treatment of *H. pylori*.

However, researches on the interactions between *C. albicans* and gut bacteria are still lacking. First, in the current studies, there are some conflicting observations, wherein the specific mechanisms are unknown. For example, although *C. albicans* can increase *S. aureus*’s resistance to antibiotics by secreting farnesol, this happens only when the concentration of farnesol is low. If the concentration of farnesol is high, its effect on *S. aureus* is opposite [203–205]. Second, most in vivo experiments were conducted using mouse, nematode, and fruit fly models, and not human ones. Finally, very little research has been conducted on the direct interactions of several bacteria with *C. albicans* in the gut.

Therefore, more number of researches on the human gut is necessary to understand, more deeply and comprehensively, the mechanisms of interactions between *C. albicans* and gut bacteria. In the future, mutant strains with deletions of specific gene fragments, vaccines, or purified biomacromolecules could serve as essential alternatives in the prevention and treatment of invasive intestinal candidiasis.

**Figure lengends**.

## Data Availability

Not applicable.

## List of abbreviations

GUT: Gastrointestinally induced transition
SPS: Ssy1p-Ptr3p-Ssy5p
HSGs: Hypha-specific genes
VAP: Ventilator-acquired pneumonia
HAI: Hospital-acquired infections
OmpA: Outer membrane protein A
ACP: Anti-*C. albicans* protein
QS: Quorum sensing
PIA: Polysaccharide intercellular adhesin
PPI: Proton pump inhibitors
AhR: Aryl hydrocarbon receptor
ILC3s: Group 3 ILCs
Treg: Regulatory T
CRAMP: Cathelicidin-related antimicrobial peptides
FMT: Fecal microbiota transplant
CDI: *C. difficile* infections
TCA: Taurocholate acidbile acid
CSP: Competence-stimulating-peptide
5MPCA: 5-methyl-phenazine-1-carboxylic acid
PYO: Pyocyanin
